# *N*-Acetyl-*L*-Cysteine as a Potential Adjunctive Strategy in STEC-HUS: Mechanistic Rationale and Current Evidence

**DOI:** 10.3390/molecules31132264

**Published:** 2026-06-29

**Authors:** Joanna Wróblewska, Marcin Wróblewski, Alina Woźniak

**Affiliations:** Department of Medical Biology and Biochemistry, Faculty of Medicine, Ludwik Rydygier Collegium Medicum in Bydgoszcz, Nicolaus Copernicus University in Toruń, 24 Karłowicza St., 85-092 Bydgoszcz, Poland; joanna.wroblewska@cm.umk.pl

**Keywords:** *Escherichia coli*, hemolytic anemia, *N*-acetyl-*L*-cysteine, oxidative stress, Shiga toxin

## Abstract

Shiga toxin-producing *Escherichia coli* (STEC) infections are a major cause of hemolytic uremic syndrome (HUS), a thrombotic microangiopathy characterized by microangiopathic hemolytic anemia, thrombocytopenia, and acute kidney injury. The pathogenesis of STEC-HUS is primarily driven by Shiga toxins (Stx), which induce endothelial injury, inflammation, platelet activation, and microvascular thrombosis. Hemolysis associated with thrombotic microangiopathy leads to the release of hemoglobin and free heme into the circulation. Free heme, an iron-containing molecule with potent pro-oxidative, pro-inflammatory, and cytotoxic properties, contributes to oxidative stress, endothelial dysfunction, complement activation, and further tissue injury. Oxidative stress plays a crucial role in both host and bacterial cells, influencing disease progression and the expression of bacterial virulence factors, including Shiga toxin. *N*-acetyl-*L*-cysteine (NAC), a precursor of glutathione (GSH) and a well-established antioxidant, has attracted attention as a potential adjunctive therapeutic agent due to its antioxidant, anti-inflammatory, antiplatelet, and cytoprotective properties. In addition, NAC may influence iron- and heme-mediated oxidative damage and improve erythrocyte resistance to oxidative stress. This review summarizes current knowledge regarding the roles of oxidative stress and free heme in STEC-HUS and examines the mechanistic rationale and current evidence supporting NAC as a potential adjunctive strategy. The available evidence remains largely indirect and preclinical; therefore, the potential role of NAC in STEC-HUS should be considered hypothesis-generating and requires further investigation in clinical studies.

## 1. Introduction

Thrombotic microangiopathies (TMA) are a group of microvascular disorders characterized by thrombus formation in small blood vessels. This process leads to the development of microangiopathic hemolytic anemia (resulting from the mechanical destruction of erythrocytes as they pass through narrowed blood vessels), thrombocytopenia, and thrombotic organ damage. Thrombotic lesions are located within the microvasculature, predominantly in the kidneys [1,2]. TMA constitutes a heterogeneous group of severe disorders that often require admission to an intensive care unit. The most common entities include thrombotic thrombocytopenic purpura (TTP), atypical hemolytic uremic syndrome (aHUS), and Shiga toxin-producing *Escherichia coli*-associated hemolytic uremic syndrome (STEC-HUS). Differentiation of TMA subtypes requires biological investigations targeted at their specific pathophysiological mechanisms. In TTP, measurement of ADAMTS13 activity is essential. In atypical hemolytic uremic syndrome (aHUS), assessment of the alternative complement pathway is particularly important [3]. The diagnosis of STEC-HUS is based on the isolation of a Shiga toxin-producing *E. coli* (STEC) strain or the detection of genes encoding Shiga toxin (*stx1*, *stx2*) directly in a stool sample [3,4]. Stool culture remains the diagnostic gold standard; however, in clinical practice, it is recommended to combine it with molecular techniques, which increase pathogen detection sensitivity and enable identification of the most important bacterial virulence factors. In cases of negative stool test results, serological diagnostics may be useful for detecting antibodies against lipopolysaccharide and STEC virulence factors, such as Stx1 and Stx2, intimin, and the translocated intimin receptor. The negative stool test result does not exclude STEC infection, as the shedding of bacteria and Stx from the gastrointestinal tract decreases over time following the onset of symptoms [4].

The natural reservoir of these bacteria is primarily the digestive tract of ruminants, especially cattle, in which they may constitute an element of the physiological intestinal microbiota. Human infection most often occurs through the consumption of contaminated food, especially undercooked beef, unpasteurized dairy products, contaminated water, and raw vegetables and fruits contaminated with animal fecal matter. *E. coli* serotype O157:H7 is most commonly associated with food- and waterborne outbreaks worldwide. [5].

Infections with STEC strains are often asymptomatic. It is estimated that approximately 75% of infected individuals do not develop any clinical symptoms [6]. The incubation period of the disease is usually one to 10 days, with symptoms most often developing within the first few days after infection [6,7]. The clinical presentation of STEC infection varies with patient age. In children, diarrhea occurs in approximately 95% of cases, and in approximately 60% of cases, it is bloody [7]. In adults, the predominant presentation is non-bloody diarrhea, occurring in approximately 83% of patients, whereas bloody diarrhea is observed in about 17% of cases [3]. During the initial phase of infection and the early stages of HUS, hemoglobin concentrations often remain within the normal range. They may even appear falsely elevated due to hemoconcentration associated with dehydration and fluid loss from diarrhea [2,8,9]. Laboratory markers of erythrocyte hemolysis, such as elevated lactate dehydrogenase (LDH) activity, decreased haptoglobin levels, and the presence of schistocytes, typically emerge several days after the onset of gastrointestinal symptoms, in parallel with the development of TMA and kidney injury [2,8]. Gastrointestinal manifestations are frequently accompanied by nausea and vomiting [3,7]. Among patients with symptomatic STEC infection, hemolytic uremic syndrome develops in approximately 15% of children and 17% of adults [6]. As the disease progresses, the characteristic features of TMA become apparent, including microangiopathic hemolytic anemia with schistocytes, thrombocytopenia, and acute kidney injury. Some patients may also develop extrarenal manifestations, particularly neurological symptoms such as headache, confusion, altered consciousness, and seizures [3,6,7].

Early diagnosis of the disease and prompt initiation of supportive therapy are essential to reduce the risk of irreversible kidney damage [10]. Supportive management includes correction of fluid and electrolyte imbalances, monitoring of hematological parameters, and renal replacement therapy when required. No uniform treatment regimen for STEC-HUS has been developed to date, and the effectiveness of many interventions used remains the subject of research [11,12]. In patients with severe disease, treatment approaches have included plasma exchange, glucocorticosteroids, and eculizumab [12]. Antibiotic therapy is not a standard part of the treatment of STEC infections, as some antibiotics, especially β-lactams and trimethoprim/sulfamethoxazole, have been shown to enhance Stx release and increase the risk of complications. At the same time, the potentially beneficial effects of macrolides, which may limit toxin synthesis, have been described [10]. Despite these controversies, observational data from the 2011 *E. coli* O104 outbreak suggested that, in patients with established STEC-HUS, antibiotic treatment was not associated with worsening of the disease course and was associated with bacterial clearance, a shorter duration of illness, and a lower frequency of seizures [12]. These findings should not be interpreted as a general recommendation for antibiotic therapy in STEC infection, as the effects may depend on the infecting strain, disease stage, and antimicrobial agent used.

Oxidative stress plays an important role in endothelial injury. Excessive production of reactive oxygen species (ROS), resulting from an imbalance between their generation and antioxidant defense mechanisms, leads to damage of lipids, proteins, and nucleic acids. Activated neutrophils represent an important source of ROS, and excessive ROS generation contributes to endothelial dysfunction and tissue injury [13]. Consequently, therapies aimed at restoring the oxidant–antioxidant balance have attracted considerable interest. *N*-acetyl-*L*-cysteine (NAC), a derivative of L-cysteine, exerts antioxidant effects by directly scavenging ROS and replenishing glutathione (GSH), one of the major endogenous antioxidants [13]. The antioxidant effects of NAC may help protect erythrocytes against oxidative damage and favorably influence hematological parameters [14]. In addition, NAC exhibits antiplatelet activity by reducing the size and activity of high-molecular-weight von Willebrand factor multimers, thereby limiting platelet adhesion and aggregation [15,16]. This antioxidant also exhibits anti-inflammatory properties, partly by reducing the production of pro-inflammatory cytokines such as interleukin-6 (IL-6) and tumor necrosis factor alpha (TNF-α), which are involved in the initiation and maintenance of the inflammatory response [17].

Due to its ability to modulate processes associated with oxidative stress, inflammation, and endothelial function, NAC is a potential adjunctive therapeutic agent in diseases characterized by microvascular injury, including STEC-HUS.

## 2. Methodology

A narrative literature review was conducted to evaluate the potential role of *N*-acetyl-*L*-cysteine (NAC) as an adjunctive therapeutic strategy in Shiga toxin-producing *Escherichia coli*–associated hemolytic uremic syndrome (STEC-HUS), with particular emphasis on oxidative stress, hemolysis, free heme toxicity, and mechanisms of endothelial injury.

The literature search was performed using the PubMed, Scopus, and Web of Science databases. Publications available up to June 2026 were considered. The search strategy combined Medical Subject Headings (MeSH) terms and free-text keywords, including: “*Escherichia coli*”, “STEC”, “Shiga toxin”, “hemolytic uremic syndrome”, “STEC-HUS”, “hemolytic anemia”, “oxidative stress”, “reactive oxygen species”, “free heme”, “hemoglobin”, “iron metabolism”, “*N*-acetyl-*L*-cysteine”, “NAC”, “glutathione”, “endothelial injury”, “inflammation”, “complement activation”, and “thrombotic microangiopathy”.

Original research articles, experimental studies, animal studies, clinical studies, observational studies, systematic reviews, and relevant review articles published in English were considered eligible. Publications focusing on the pathogenesis of STEC infection, oxidative stress mechanisms, heme- and iron-mediated tissue injury, antioxidant therapies, and the biological effects of NAC were prioritized. Conference abstracts lacking sufficient methodological details and publications unavailable in full text were also excluded when critical information could not be verified. Relevant studies were screened based on titles and abstracts, followed by full-text evaluation when appropriate. Information concerning the pathogenesis of STEC-HUS, the role of oxidative stress and free heme, mechanisms of iron-mediated tissue injury, and the antioxidant, anti-inflammatory, and antithrombotic properties of NAC was extracted and synthesized narratively. Due to the lack of clinical studies directly evaluating the use of NAC in STEC-HUS, evidence from experimental models and studies of other conditions associated with hemolysis, kidney injury, thrombotic microangiopathies, and increased oxidative stress was also considered. This approach enabled assessment of the biological and mechanistic rationale supporting the potential use of NAC in STEC-HUS. The available evidence regarding NAC in STEC-HUS remains limited and is derived predominantly from experimental studies. Therefore, conclusions concerning the therapeutic potential of NAC should be regarded as hypothesis-generating and interpreted with caution until validated in prospective clinical studies.

## 3. The Importance of Iron and Iron Acquisition Mechanisms in Pathogenic *Escherichia coli* Strains

The ability to cause disease is associated with the presence of specific virulence factors. Depending on the virulence factors present and the host’s clinical manifestations, *E. coli* strains are classified into pathotypes: intestinal pathogenic *E. coli* (IPEC) and extraintestinal pathogenic *E. coli* (ExPEC). Within the IPEC group, diarrheagenic *E. coli* are distinguished, including enteropathogenic, enterotoxigenic, enteroinvasive, enteroaggregative, diffusely adherent, and enterohemorrhagic (EHEC) strains [18]. EHEC strains play a significant role in the pathogenesis of diarrhea-associated HUS, which is the most common cause, with serotype O157 being the predominant serotype worldwide in this group [19]. The key virulence factor of EHEC strains that plays a central role in the development of HUS is Stx [19]. Strains that produce Stx are collectively referred to as STEC and include both EHEC strains and other variants capable of producing Stx [20]. Stx plays a crucial role in vascular endothelial cell injury by initiating a cascade that leads to the development of TMA, characterized by the classical triad of hemolytic anemia, thrombocytopenia, and acute kidney injury. Hemolysis of erythrocytes results in the release of hemoglobin and free heme into the circulation. The observed clinical variability of the disease suggests the involvement of additional factors that exacerbate endothelial damage. One such factor may be free heme, which exhibits pro-oxidative, pro-inflammatory, and prothrombotic properties and may aggravate the vascular injury observed in STEC-HUS [21].

The release of hemoglobin and heme during hemolysis is important not only for the pathogenesis of STEC-HUS but also for iron homeostasis. Heme represents an important form of iron in the body, and the iron released during hemolysis is tightly regulated in order to limit its toxic effects and its availability to microorganisms. Maintaining appropriate iron levels is essential for preserving iron homeostasis and ensuring the proper functioning of numerous biological processes. Elevated iron concentrations are associated with various pathophysiological disorders, including neurodegenerative diseases, cancer, endocrine disorders, diabetes, liver disease, cardiovascular disease, and impaired immune system function. Conversely, iron deficiency may lead to anemia as well as impaired activation and proliferation of immune cells, highlighting the importance of maintaining iron balance within the body [22]. Iron acts as a biocatalyst or cofactor in numerous chemical reactions occurring within the cell. It is involved in oxygen binding and transport, nitrogen fixation, hydrogen (H_2_) production and utilization, cellular respiration, the functioning of the tricarboxylic acid cycle (Krebs cycle), regulation of gene expression, and DNA biosynthesis [23].

The availability of iron to pathogenic bacteria is limited because the host binds and sequesters this element through proteins such as transferrin, lactoferrin, and ferritin. This mechanism constitutes an important component of the antimicrobial defense response and is referred to as nutritional immunity, which involves restricting the availability of essential micronutrients to pathogens [22,23,24]. In response to limited iron availability, bacteria have evolved specialized mechanisms that enable them to acquire this element from the host environment. In ExPEC, siderophore systems are considered particularly important. Siderophores are small molecules with a high affinity for iron that are secreted outside the cell, bind Fe^3+^, and are subsequently transported back into the bacterial cell as ferri-siderophore complexes [24]. The ability to efficiently acquire iron constitutes an important virulence-associated trait of *E. coli*. An alternative iron acquisition strategy involves the utilization of heme present in host hemoproteins, such as hemoglobin and hemopexin [25]. *E. coli* expresses specific outer membrane receptors that bind host heme-containing proteins or secretes hemophores (heme-binding proteins), which subsequently deliver the heme–hemophore complex to receptors located on the bacterial cell surface [23]. Pathogenic *E. coli* strains, including EHEC, can utilize heme and hemoglobin as iron sources, enabling their growth under iron-limited conditions. This mechanism may involve the outer membrane transporter ChuA, which binds host hemoglobin and mediates heme extraction and import into the bacterial cell. Hemoglobin has been shown to be a high-affinity substrate for ChuA, and its availability may support bacterial growth in iron-restricted environments [26,27]. These bacteria may also induce hemolysis, leading to erythrocyte lysis and the release of hemoglobin and heme, which can subsequently be utilized as sources of iron [23]. In the context of STEC-HUS, these mechanisms may acquire additional relevance because microangiopathic hemolysis is associated with the release of hemoglobin and free heme into the circulation [21,28]. Given that pathogenic *E. coli* possess dedicated heme acquisition systems, increased heme availability may provide an additional source of iron for bacterial uptake [23,25].

## 4. Role of Shiga Toxins in the Pathogenesis of Hemolytic Uremic Syndrome

Stx is the main virulence factor of STEC strains. Two major families of these toxins are distinguished: Stx1 (including the Stx1, Stx1c, and Stx1d variants) and Stx2 (including, among others, the Stx2c, Stx2c2, Stx2d, Stx2_dactivatable_, Stx2e, and Stx2f variants) [29]. The clinical significance of individual types is not equivalent. Stx2, Stx2c, and Stx2_dactivatable_ are associated with the development of hemorrhagic colitis and HUS, whereas the remaining variants are associated with asymptomatic infections or uncomplicated diarrhea [29]. Genes encoding Stx are not native to the *E. coli* genome but are derived from lambdoid bacteriophages, known as Shiga toxin-converting phages [20]. Following infection of a bacterial cell, the phage genome integrates into the host chromosome as a prophage, rendering the bacterium a carrier of *stx* genes [20,30]. Shiga toxin-encoding phages may infect not only *E. coli* but also certain commensal intestinal bacteria, thereby facilitating the further dissemination of *stx* genes [30].

Stx possesses a characteristic AB_5_ structure composed of one enzymatically active A subunit and five identical B subunits, which are responsible for recognition and binding to the cellular receptor [30]. The primary receptor for Stx is globotriaosylceramide (Gb3Cer, Gb3), which is predominantly located within lipid rafts of the cell membrane [29,30]. The presence of the Gb3 on endothelial cells, particularly those of the renal and cerebral vasculature, as well as on renal tubular epithelial cells, is associated with the high susceptibility of these tissues to the effects of the toxins [31].

Following binding to the Gb3 receptor, the toxin is internalized and subsequently transported through the Golgi apparatus to the endoplasmic reticulum. There, the A subunit is activated, and the resulting A1 fragment translocates to the cytoplasm, where it exhibits rRNA N-glycosidase activity and removes a single adenine residue from the 28S ribosomal RNA of the 60S ribosomal subunit, resulting in inhibition of protein synthesis [29]. Damage to ribosomal RNA by Stx induces a ribotoxic stress response, leading to activation of cellular stress-associated signaling pathways, including p38 mitogen-activated protein kinase. Consequently, the expression of pro-inflammatory mediators is increased, and cellular processes that impair cell function and may lead to cellular injury or death are activated [29,31]. Stx may bind to erythrocytes, platelets, and monocytes, which express receptors for these toxins. Interactions between Stx and neutrophils have also been described, although their role in toxin transport remains a matter of debate [29]. In response to toxin activity, increased production of pro-inflammatory cytokines, such as TNF-α, IL-6, interleukin 1 beta (IL-1β), and interleukin 8 (IL-8), occurs, exacerbating endothelial damage and promoting the development of TMA [29]. During STEC-HUS, Stx-induced endothelial injury and microangiopathic hemolysis lead to the release of hemoglobin, which can subsequently liberate free heme into the circulation. These processes contribute to oxidative stress and inflammation [28,32,33,34]. Free heme is an important source of redox-active iron that participates in the Fenton reaction, leading to the generation of toxic hydroxyl radicals and increased cellular damage [35]. In response to infection, hepcidin, a hormone that regulates iron metabolism, is induced, leading to iron sequestration and a rapid decrease in its concentration in plasma and extracellular fluids (hypoferremia). This mechanism constitutes a component of the host defense response that limits iron availability to microorganisms [36]. Simultaneously, Stx induces platelet activation, adhesion, and aggregation within damaged endothelium, promoting the formation of microthrombi and disturbances in microcirculation. Increased platelet consumption within these lesions contributes to the development of thrombocytopenia, a hallmark feature of HUS [37].

## 5. Mechanisms of Oxidative Stress and Inflammation

Oxidative stress is an important factor causing damage to bacterial cells. ROS are generated mainly intracellularly as by-products of aerobic metabolism. Their presence leads to damage of iron-containing enzymes and genetic material, which may disrupt metabolic pathways and increase mutation frequency. Severe oxidative stress is also associated with impaired bacterial growth and, in extreme cases, may lead to cell death [38]. In mammalian cells, ROS affect protein function and regulate signaling pathways by activating transcription factors and altering gene expression. As a result, they activate pro-inflammatory pathways, leading to endothelial activation, increased leukocyte recruitment, and enhanced interactions between leukocytes, platelets, and the endothelium. These processes contribute to the development of vascular injury, particularly under hemolytic conditions [34]. Increased ROS production further impairs endothelial function and enhances the activation of pro-inflammatory pathways, promoting leukocyte recruitment and the development of vascular damage [17,39]. This process supports pathogen elimination; however, excessive or sustained activation of the inflammatory response may simultaneously exacerbate tissue damage and perpetuate inflammation [39].

In STEC-HUS, both the inflammatory response and oxidative stress are intensified, mutually reinforcing one another and contributing to the development of endothelial and renal injury [13,21,39]. During the inflammatory response, ROS production increases, contributing to pathogen elimination and regulation of immune responses. However, excessive ROS generation leads to damage of proteins, lipids, and DNA, resulting in tissue dysfunction and further amplification of vascular injury [32]. Experimental studies have demonstrated that both Stx and STEC-produced hemolysin enhance ROS generation in blood cells, which is associated with protein oxidation, increased levels of advanced oxidation protein products, and apoptosis. A biphasic relationship between toxin concentration and ROS production has also been observed. The greatest increase in oxidative stress was detected following exposure to 200 μg of Stx and 0.2 HU of hemolysin. In contrast, higher toxin concentrations were associated with increased cell death and a secondary reduction in ROS production [40]. Furthermore, Stx2 enhances ROS production in the host, particularly in the kidneys, accompanied by decreased GSH levels and increased lipid peroxidation in renal tissue, indicating membrane damage and disruption of the oxidant-antioxidant balance [13]. At the same time, patients with STEC-HUS exhibit increased plasma total antioxidant capacity, indicating activation of endogenous defense mechanisms against oxidative stress. Enhanced plasma antioxidant activity reduces ROS production by blood cells, suggesting a potential protective role during the course of the disease [40].

The inflammatory response also plays a significant role in the pathogenesis of HUS. Cytokines such as TNF and IL-1 may be produced by renal cells in response to Stx exposure. These cytokines act synergistically with Stx, enhancing its cytotoxic effects on cells, including endothelial cells, in part by increasing the expression of toxin receptors. In addition, they amplify processes leading to cellular injury and apoptosis [19]. Among inflammatory mediators involved in HUS pathogenesis, particular attention has been given to TNF-α, IL-6, IL-8, and granulocyte colony-stimulating factor (G-CSF). These mediators participate in the recruitment and activation of neutrophils and monocytes, which are involved in disease-associated inflammatory processes. Activated neutrophils release ROS, proteases, and neutrophil extracellular traps (NETs), which may contribute to endothelial injury. NETs also promote platelet adhesion, activation, and aggregation, as well as fibrin deposition, thereby serving as an important link between inflammation and thrombosis [41]. Patients with STEC-HUS exhibit elevated levels of TNF-α, IL-6, IL-8, and G-CSF compared with healthy individuals. Higher concentrations of TNF-α and IL-6 correlate with a more severe disease course and the occurrence of extrarenal complications, whereas elevated levels of IL-6 and soluble TNF receptor type I are associated with encephalopathy [41].

Mechanical hemolysis of erythrocytes results in the release of hemoglobin into the circulation, the oxidation of which may lead to the liberation of free heme. As an iron-containing molecule, free heme exhibits potent pro-oxidative and cytotoxic properties, contributing to tissue injury and amplification of inflammatory responses [33]. Owing to its hydrophobic nature, free heme may become incorporated into cellular membranes, increasing their susceptibility to oxidative damage [34]. Furthermore, free heme promotes complement activation, induces endothelial adhesion molecule expression, and enhances leukocyte adhesion and platelet activation, thereby contributing to vascular inflammation and injury [28]. In response, the host activates both extracellular mechanisms responsible for binding and removing heme and hemoglobin, involving proteins such as haptoglobin, hemopexin, albumin, high-density lipoproteins, low-density lipoproteins (LDL), and α1-microglobulin, as well as intracellular mechanisms associated with the induction of heme oxygenase-1 and sequestration of released iron by ferritin [34]. These mechanisms limit oxidative stress exacerbated by free hemoglobin and heme, which can initiate oxidative reactions and cellular injury [42]. In addition, haptoglobin binds free hemoglobin, limiting heme release, whereas hemopexin binds and neutralizes free heme. Exhaustion of these protective mechanisms results in increased free heme levels, which may contribute to increased oxidative stress, inflammation, and kidney injury. Patients with STEC-HUS have been reported to exhibit reduced levels of haptoglobin and hemopexin together with increased concentrations of free heme [28]. Stx2-induced complement-mediated hemolysis is associated with the release of hemoglobin and LDH. Patients with STEC-HUS exhibit increased numbers of erythrocyte-derived microvesicles carrying complement components such as C3, C9, and C5b-9. Because these microvesicles expose phosphatidylserine, they may further enhance the prothrombotic state characteristic of STEC-HUS [43]. Limiting heme availability also serves as a defense mechanism against pathogens by reducing iron availability. It has been shown that interleukin-22 (IL-22) induces the production of plasma proteins that bind hemoglobin and heme, particularly hemopexin and haptoglobin. IL-22 deficiency is associated with increased bacterial proliferation and higher mortality during systemic bacterial infections, as demonstrated in murine models of *E. coli* infection [44].

## 6. Redox Properties of *N*-Acetyl-*L*-Cysteine and Its Interactions with Heme and Iron

**Figure 1:**
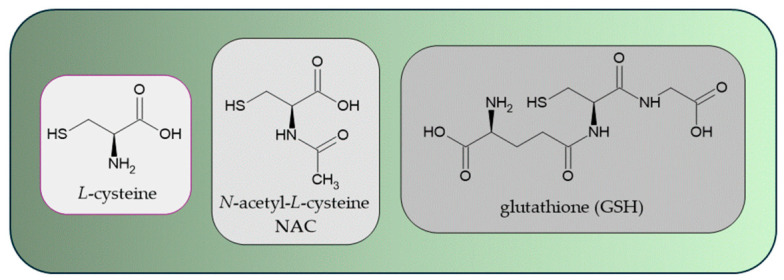
Structural relationship between *L*-cysteine, *N*-acetyl-*L*-cysteine (NAC), and glutathione (GSH).

**Figure 2:**
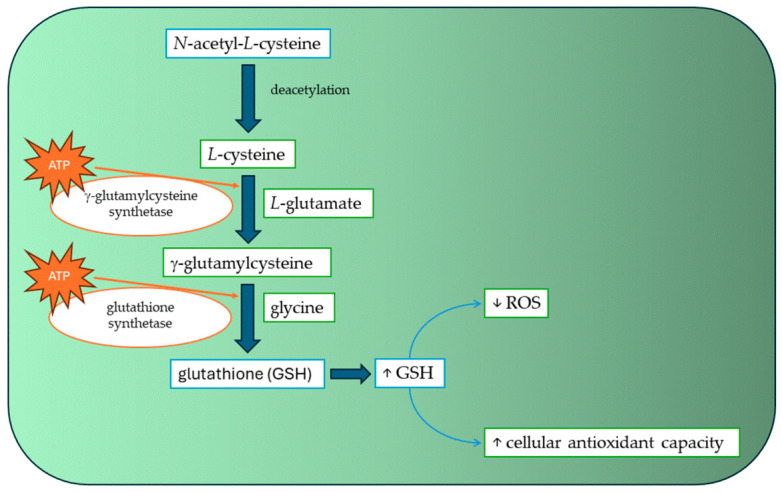
Contribution of *N*-acetyl-*L*-cysteine to intracellular glutathione biosynthesis. Following deacetylation to *L*-cysteine, NAC provides the rate-limiting substrate for glutathione synthesis, resulting in increased cellular antioxidant capacity and reduced reactive oxygen species (ROS) levels. (↑ increase, ↓ decrease).

NAC is the *N*-acetyl derivative of the naturally occurring amino acid *L*-cysteine. The structural relationship between *L*-cysteine, NAC, and glutathione is shown in Figure 1. Its molecular structure comprises a carboxyl group, an acetamide moiety, and a reactive thiol (-SH) group, which is primarily responsible for its antioxidant and nucleophilic properties. NAC possesses the molecular formula C_5_H_9_NO_3_S and a molecular weight of 163.19 g/mol. Acetylation of the amino group enhances the chemical stability of cysteine while maintaining the biological reactivity associated with the sulfhydryl functionality [45]. The physicochemical behavior of NAC is strongly influenced by its acid-base properties. The carboxyl group exhibits a pKa of approximately 3.24, whereas the thiol group has a pKa of approximately 9.52. Consequently, at physiological pH (7.4), the carboxyl group is predominantly deprotonated, while the thiol group remains largely protonated. Under these conditions, NAC exists mainly as an anionic species, which significantly affects its absorption, distribution, and membrane permeability [46,47]. NAC is characterized by low lipophilicity, with reported logP values ranging from approximately −0.4 to −3.4. Due to its ionization at physiological pH, the distribution coefficient (logD_7.4_) is even lower, reflecting its predominantly hydrophilic nature. The topological polar surface area (tPSA) of NAC is approximately 92 Å^2^, and the molecule contains several hydrogen-bond donor and acceptor sites. These molecular descriptors contribute to excellent aqueous solubility but limit passive diffusion across biological membranes, thereby contributing to the relatively low oral bioavailability reported for NAC [48,49]. The thiol group constitutes the principal redox-active center of NAC. Through reversible oxidation, NAC can form disulfides, including *N*,*N*′-diacetylcystine and mixed disulfides with endogenous thiol-containing molecules. The sulfhydryl group acts as a nucleophile capable of reacting with electrophilic oxidants and ROS. Although the direct free radical scavenging capacity of NAC is moderate compared with endogenous antioxidant systems, its ability to participate in thiol-disulfide exchange reactions contributes substantially to cellular redox regulation and protection against oxidative stress [46,50]. A major mechanism underlying the biological activity of NAC involves its role as a precursor of intracellular cysteine and GSH. Following cellular uptake and deacetylation, NAC supplies cysteine, the rate-limiting substrate for GSH biosynthesis. The role of NAC in glutathione biosynthesis is illustrated in Figure 2. Increased intracellular GSH concentrations support detoxification pathways mediated by glutathione peroxidases and glutathione S-transferases, facilitate the reduction of peroxides, and maintain cellular redox homeostasis. Consequently, many of the antioxidant and cytoprotective effects of NAC are attributed indirectly to enhanced GSH synthesis rather than direct radical scavenging activity [50,51]. The pharmacological profile of NAC is closely linked to its structure-property relationships. The acetyl group improves chemical stability and pharmaceutical applicability relative to L-cysteine, while the thiol functionality confers antioxidant, reducing, and mucolytic properties. Conversely, the high polarity, low lipophilicity, and ionization state at physiological pH restrict passive membrane permeation and contribute to limited oral bioavailability. Therefore, the therapeutic efficacy of NAC results from the interplay between its molecular structure, physicochemical characteristics, thiol-mediated redox chemistry, and central role in GSH metabolism [45,48,50,51].

**Figure 3:**
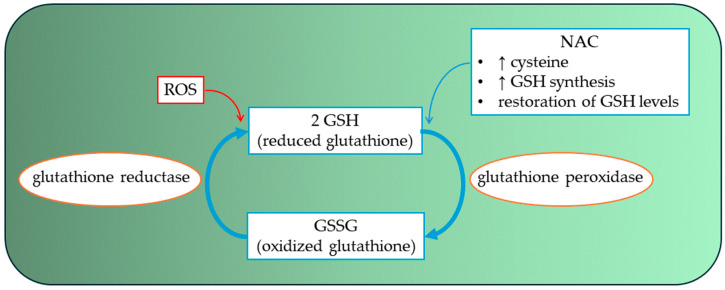
Effect of *N*-acetyl-*L*-cysteine (NAC) on glutathione redox cycling and antioxidant defense. NAC replenishes intracellular cysteine and supports glutathione synthesis, promoting restoration of reduced glutathione (GSH) levels and enhancing cellular antioxidant capacity through the glutathione peroxidase/glutathione reductase system. ROS—reactive oxygen species. (↑ increase).

Free heme and redox-active iron are important contributors to oxidative injury in numerous pathological conditions. Following hemolysis or tissue damage, the release of heme from hemoproteins increases the availability of catalytic iron, which can participate in redox cycling and promote the formation of reactive oxygen species (ROS). In particular, ferrous iron (Fe^2+^) reacts with hydrogen peroxide through the Fenton reaction, generating highly reactive hydroxyl radicals (•OH), whereas ferric iron (Fe^3+^) can be reduced back to Fe^2+^ by biological reductants, sustaining oxidative damage through continuous redox cycling [52,53]. NAC may influence heme- and iron-mediated oxidative stress through several interconnected chemical mechanisms. First, the thiol (-SH) group of NAC acts as a reducing agent capable of participating in thiol-disulfide exchange reactions and maintaining intracellular redox balance (Figure 3) [50]. Although NAC itself may reduce Fe^3+^ to Fe^2+^ under certain conditions, potentially supporting iron redox cycling, its overall biological effect is generally antioxidant because it simultaneously decreases the availability of reactive oxygen species required to sustain oxidative chain reactions [45,50]. A second major mechanism involves replenishment of intracellular GSH. Following deacetylation to cysteine, NAC provides the rate-limiting substrate for GSH biosynthesis [45,51]. Increased GSH concentrations facilitate the detoxification of hydrogen peroxide through glutathione peroxidase-catalyzed reactions, thereby decreasing the concentration of H_2_O_2_ available for Fenton chemistry [51]. As a result, the generation of hydroxyl radicals from iron-catalyzed reactions is indirectly reduced. In addition, GSH and other low-molecular-weight thiols contribute to maintaining iron homeostasis by modulating the cellular redox environment and protecting proteins from oxidative modification [50]. NAC-mediated restoration of intracellular thiol pools may therefore attenuate oxidative damage associated with free heme and labile iron accumulation. Experimental studies have demonstrated that NAC can reduce lipid peroxidation, protein oxidation, and oxidative tissue injury in models characterized by excessive iron-dependent ROS production [45,50]. The relationship between NAC and iron chemistry is complex because thiol-containing molecules can exert both antioxidant and pro-oxidant effects depending on experimental conditions, metal concentrations, and oxygen availability [50]. While reduction of Fe^3+^ to Fe^2+^ may theoretically favor Fenton reactions, the predominant biological consequence of NAC administration appears to be suppression of oxidative stress through enhancement of glutathione-dependent antioxidant defenses, scavenging of reactive oxidants, and limitation of peroxide accumulation [45,51]. Consequently, NAC is generally considered to mitigate rather than promote heme- and iron-mediated oxidative injury.

**Figure 4:**
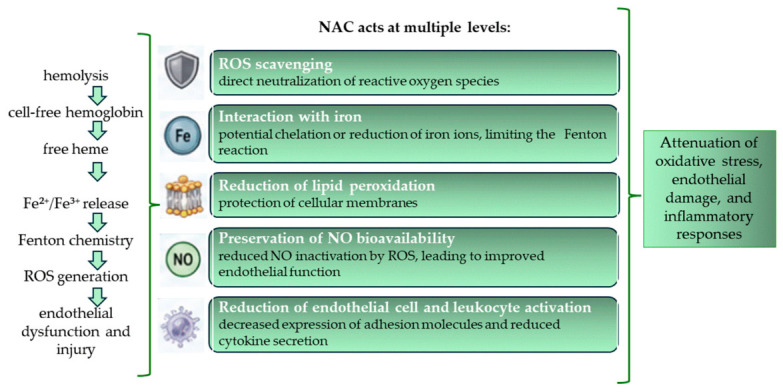
Dual effects of *N*-acetyl-*L*-cysteine on iron-dependent redox reactions. NO—nitrogen oxide, ROS—reactive oxygen species.

Although NAC is widely recognized for its antioxidant properties, its behavior in the presence of redox-active transition metals is more complex. Thiol-containing compounds can act as reducing agents and may reduce ferric iron (Fe^3+^) to ferrous iron (Fe^2+^), thereby potentially sustaining iron redox cycling [46,50]. Because Fe^2+^ is the catalytically active form of iron in the Fenton reaction, such reduction could theoretically increase hydroxyl radical (•OH) generation in environments characterized by elevated concentrations of both free iron and hydrogen peroxide [52]. Under severe hemolytic conditions, substantial amounts of free heme and non-transferrin-bound iron may be released into the extracellular milieu. In such settings, high local concentrations of low-molecular-weight thiols, including NAC, could in principle contribute to the reduction of Fe^3+^ to Fe^2+^, thereby enhancing the availability of redox-active iron. This mechanism has been demonstrated in simplified chemical systems and may lead to apparent pro-oxidant effects under specific experimental conditions [46]. However, the biological relevance of this phenomenon remains uncertain. In living systems, NAC simultaneously increases intracellular GSH synthesis, supports glutathione peroxidase activity, and promotes the detoxification of hydrogen peroxide and lipid hydroperoxides [45,51]. Since hydrogen peroxide is a required substrate for hydroxyl radical generation through Fenton chemistry, depletion of peroxide generally limits oxidative injury despite potential enhancement of Fe^3+^ reduction. Consequently, the net effect of NAC administration is usually antioxidant rather than pro-oxidant [50]. Therefore, while NAC may theoretically promote Fe^3+^/Fe^2+^ cycling under conditions of excessive iron availability, the overall balance between pro-oxidant and antioxidant effects depends on iron concentration, peroxide availability, oxygen tension, thiol concentration, and the efficiency of endogenous antioxidant systems. Current evidence suggests that GSH replenishment and peroxide detoxification predominate in most biological contexts, resulting in a net reduction in oxidative stress. The balance between the potential pro-oxidant and antioxidant effects of NAC is presented in Figure 4.

## 7. Potential Points of Action and Current Evidence for NAC in STEC-HUS

Oxidative stress constitutes an important component of HUS caused by STEC strains [40,54]. The significance of oxidative stress in the pathogenesis of STEC-HUS suggests that modulating oxidative stress may represent a potential direction for adjunctive therapy, which justifies interest in exogenous antioxidants such as NAC. Although NAC has been proposed as a potential adjunctive approach in STEC-HUS, its clinical efficacy in this condition has not yet been established. Current evidence supporting this concept is derived largely from experimental studies and indirect clinical observations in other disease settings, particularly kidney disease, whereas clinical studies evaluating NAC in STEC-HUS patients are lacking [55,56,57,58,59]. The different levels of evidence supporting the proposed role of NAC in STEC-HUS are summarized in Table 1.

**Figure 5:**
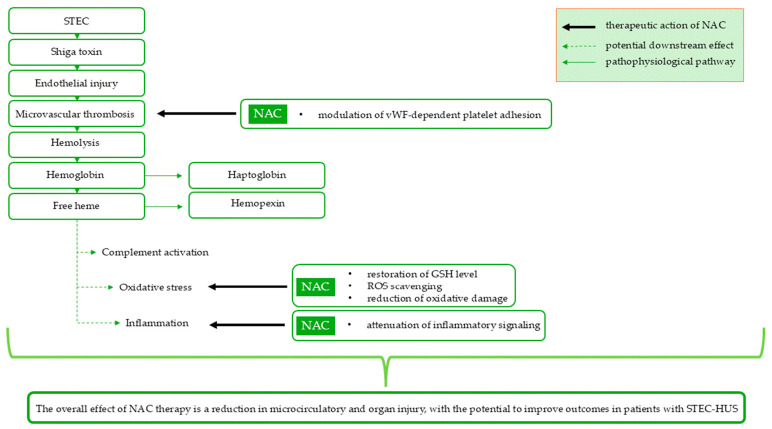
Pathogenic cascade in STEC-HUS and potential points of intervention by *N*-acetyl-*L*-cysteine.

The potential therapeutic rationale for NAC in STEC-HUS is based primarily on three interconnected mechanisms: reduction in oxidative stress, protection against hemolysis-related oxidative injury, and modulation of inflammatory pathways. The proposed sites of NAC action within the pathogenic network of STEC-HUS are summarized in Figure 5.

NAC exhibits pleiotropic antioxidant activity through modulation of GSH-dependent antioxidant pathways and direct thiol-mediated redox reactions [60,61]. In addition to its effects on glutathione homeostasis, NAC may modulate the activity of the transcription factor nuclear factor erythroid 2-related factor 2, which regulates the expression of numerous genes involved in the cellular antioxidant response [56]. NAC may also influence protein structure and function by reducing disulfide bonds between cysteine residues, thereby altering their conformation and biological properties [60].

Beyond its effects on GSH homeostasis, NAC has been shown to protect erythrocytes against oxidative damage by modulating the activity of reductive enzymes. NAC supplementation was associated with increased activity of methemoglobin reductase and ferricyanide reductase, which may indicate improved functioning of antioxidant defense mechanisms, protection of the erythrocyte membrane against lipid peroxidation, and prolonged survival of red blood cells [14]. These observations suggest that NAC may attenuate hemolysis-related oxidative injury, one of the key pathological features of STEC-HUS.

Additional observations from studies conducted in patients with chronic kidney disease suggest that NAC may influence erythropoiesis. The use of NAC may also be associated with a reduction in parathyroid hormone (PTH) levels. Since elevated PTH may impair erythropoiesis, the decrease in PTH observed during NAC therapy has been linked to improved hemoglobin concentrations, suggesting a potentially beneficial effect on erythropoietic processes and a reduction in erythropoietin resistance [57]. The effect of NAC on anemia-related parameters remains inconclusive. Although an improvement in hemoglobin concentration has been reported following NAC supplementation, this finding has not been consistently confirmed [58,59]. These discrepant results may be related, at least in part, to differences in treatment duration. A short treatment period may be insufficient to achieve significant improvement in hematological parameters, which could explain the lack of a significant increase in hemoglobin concentration observed in studies with short-duration therapy [59].

NAC also exhibits anti-inflammatory properties. It may reduce levels of pro-inflammatory cytokines such as TNF-α, IL-6, and IL-1β, among other mechanisms, by inhibiting the transcription factor nuclear factor kappa B, which plays a key role in regulating the inflammatory response [55]. The anti-inflammatory effects of NAC are also reflected in changes in laboratory markers. Since ferritin functions both as an iron-storage protein and an acute-phase protein, the reduced ferritin concentrations observed following NAC administration may indirectly reflect attenuation of inflammation and oxidative stress [57].

In addition to its antioxidant and anti-inflammatory activities, NAC has also been shown to exhibit antimicrobial properties [62,63,64]. Its interaction with antibiotics appears to be complex and antibiotic-specific. In vitro studies in *E. coli* have reported both enhancement (e.g., fluoroquinolones and amikacin) and attenuation (e.g., streptomycin, rifampicin, and ceftriaxone) of antibacterial activity in the presence of NAC [62]. Although the potential relevance of these findings to STEC-HUS remains to be established, experimental in vitro studies suggest that NAC may reduce bacterial adhesion and aggregation and impair biofilm formation [63,64,65].

An experimental study demonstrated that administering exogenous antioxidants, such as NAC and *S*-ethyl-*L*-cysteine (SEC), may partially mitigate the effects of Stx-induced oxidative stress. These compounds reduced ROS production, attenuated renal injury, and improved survival in an animal model. The authors suggested that early initiation of antioxidant therapy, already during the prodromal phase or the diarrheal stage, could help maintain oxidative-antioxidative balance and limit tissue damage [13]. NAC appears to be most effective when administered before or during the early phase of exposure to a tissue-damaging factor [56,66]. Delayed administration may be less beneficial because, in the setting of established tissue injury and inflammation, a paradoxical increase in oxidative stress, potentially driven by the compound’s pro-oxidant activity, has been observed [66]. In an experimental model of folic acid-induced acute kidney injury, NAC administered before the onset of injury reduced oxidative stress, increased GSH levels, and attenuated renal damage, whereas administration after injury induction did not provide comparable benefits [66]. However, no prospective clinical studies have evaluated NAC administration at different stages of STEC-HUS. Therefore, despite experimental evidence suggesting that earlier administration may be more beneficial, no evidence-based recommendations can currently be made regarding the optimal therapeutic window. Available data do not allow determination of whether NAC should be administered during the prodromal diarrheal phase, after microbiological confirmation of STEC infection, at the onset of microangiopathic hemolysis, or only after established HUS has developed. Consequently, NAC should currently be regarded as an investigational adjunctive strategy in STEC-HUS pending further clinical evaluation of both its efficacy and the optimal timing of administration.

A limitation of NAC is its short half-life and low bioavailability, which necessitate the use of higher and more frequent dosing regimens [61]. The half-life of NAC is approximately 6.25 h. Its elimination occurs through both renal and non-renal pathways [60]. Safety considerations regarding NAC in STEC-HUS remain insufficiently defined. However, available experience from other thrombotic microangiopathies has not identified major safety concerns associated with NAC administration, although the available safety data remain limited and are derived primarily from case reports and observational studies [67]. The most commonly reported adverse effects involve the gastrointestinal tract and include nausea, vomiting, and diarrhea [60]. It has been demonstrated that liposomal administration of NAC enables its gradual release, prolongs circulation time, and improves bioavailability. Liposomal NAC was more effective than free NAC in protecting renal cells against iron overload-induced injury and oxidative stress under in vitro conditions [61]. In cases of complications related to NAC overdose, management primarily involves immediate discontinuation of NAC administration and supportive treatment, including renal replacement therapy in severe cases. In a case report of aHUS induced by NAC overdose, favorable outcomes were achieved following treatment with eculizumab, an inhibitor of complement component C5 [68].

**Table 1:** Levels of evidence supporting the proposed role of NAC in STEC-HUS.

Evidence Category	Study Characteristics and Key Findings	Potential Relevance to STEC-HUS	Ref.
Experimental STEC-HUS evidence	Murine Stx2-induced HUS model evaluating the role of oxidative stress and antioxidant therapy (↓ MDA, attenuated ROS generation by PMN, ↓ platelet activation, ↓ renal injury, ↓ plasma urea, ↑ survival following NAC treatment)	Direct experimental evidence. In a murine Stx2-induced HUS model relevant to STEC-HUS, NAC reduced oxidative stress and renal injury and improved survival	[13]
In vitro studies	Human kidney cell model of iron overload-induced toxicity evaluating the protective effects of NAC and liposomal NAC (↑ cell viability, ↓ ROS generation, protection against iron-induced cytotoxicity; enhanced efficacy with liposomal NAC)	Indirect experimental evidence. NAC reduced ROS generation and protected human renal tubular cells from oxidative injury in an iron-overload model, supporting the concept that antioxidant strategies may help limit oxidative stress–associated renal injury relevant to STEC-HUS	[61]
Clinical studies in CKD/hemodialysis patients	Clinical study in hemodialysis patients evaluating the effects of oral NAC on oxidative stress and anemia (↑ Hct, ↑ RBC reductase activities, ↑ TAC, ↓ 8-izoprostan, ↓ ox-LDL)	NAC improved oxidative stress and anemia-related parameters in hemodialysis patients, supporting the biological plausibility that modulation of oxidative stress may influence mechanisms relevant to STEC-HUS	[14]
	Clinical study in children with kidney failure undergoing maintenance hemodialysis evaluating the effects of oral NAC on oxidative stress, anemia and cardiac function (↑ hemoglobin concentration, ↓ ferritin level, ↑ TAC, ↓ TOS, ↓ PTH)		[57]
	Clinical study in hemodialysis patients evaluating the effects of oral NAC on inflammation and anemia-related parameters (↓ hs-CRP, ↑ hemoglobin, ↑ serum iron, ↑ transferrin saturation, no significant changes in MDA and ferritin levels were observed)	Indirect clinical evidence. NAC improved anemia-related parameters and reduced systemic inflammation in hemodialysis patients, supporting potential relevance to inflammatory and hematologic processes involved in STEC-HUS	[58]
	Clinical study in patients with end-stage renal disease (↓ hs-CRP, ↓ ferritin levels, ↓ Hct, no significant differences: hemoglobin levels)	Indirect clinical evidence. NAC reduced inflammatory markers in hemodialysis patients, but did not improve anemia; this supports potential relevance mainly to inflammatory, rather than hematologic, processes involved in STEC-HUS.	[59]
Mechanistic hypotheses	Narrative review summarizing the antioxidant and anti-inflammatory mechanisms of NAC	Mechanistic support. NAC may counteract oxidative stress and inflammation through glutathione restoration and suppression of pro-inflammatory signaling pathways implicated in STEC-HUS.	[55]

CKD—Chronic Kidney Disease; Hct—average of hematocrit; hs-CRP—high-sensitive C-reactive protein; MDA—malondialdehyde; NAC—*N*-acetyl-*L*-cysteine; PMN—polymorphonuclear leucocytes; PTH—parathormone; RBC—red blood cells; TAC—total antioxidant capacity; TOS—total oxidative stress. Clinical studies in CKD/hemodialysis patients should be regarded as indirect evidence because the mechanisms underlying CKD-associated anemia differ fundamentally from STEC-HUS-related microangiopathic hemolytic anemia, ↑—increase, ↓—decrease.

## 8. Conclusions

Despite promising experimental findings, the role of NAC in STEC-HUS remains uncertain. Current evidence is derived predominantly from experimental models and indirect observations, whereas clinical studies evaluating NAC in STEC-HUS patients are lacking. The antioxidant, anti-inflammatory, and antiplatelet properties of NAC provide a mechanistic rationale for further investigation of this compound in STEC-HUS. In addition, experimental studies have shown that NAC may inhibit platelet activation and aggregation, suggesting a potential benefit in thrombotic microangiopathies; however, their clinical relevance remains uncertain. Experimental evidence suggests that NAC may reduce oxidative stress, increase GSH levels, and attenuate endothelial and renal injury, particularly when administered during the early stages of disease development; however, there is still insufficient clinical evidence to support its routine use. A major limitation is that most available data originate from animal models, limiting the direct translation of these findings into clinical practice in humans.

Furthermore, the optimal dose, the most appropriate timing for treatment initiation, the patient populations most likely to benefit, and the optimal duration of therapy have not yet been established. Another important limitation is the possibility of a narrow therapeutic window. Although experimental studies suggest that the biological effects of NAC may depend on the timing of administration, these findings have not been confirmed in clinical studies involving patients with STEC-HUS. Therefore, the potential role of NAC in STEC-HUS should currently be regarded as hypothesis-generating. Well-designed clinical studies are needed to determine its safety, efficacy, and possible place in the management of STEC-HUS.

## Data Availability

No new data were created or analyzed in this study. Data sharing is not applicable to this article.

## Abbreviations

The following abbreviations are used in this manuscript:

aHUS	Atypical hemolytic uremic syndrome
EHEC	Enterohemorrhagic *Escherichia coli*
ExPEC	Extraintestinal pathogenic *Escherichia coli*
Gb3	Globotriaosylceramide
G-CSF	Granulocyte colony-stimulating factor
GSH	Glutathione
Hct	Hematocrit
hs-CRP	High-sensitive C-reactive protein
IL-1β	Interleukin 1-beta
IL-6	Interleukin-6
IL-8	Interleukin-8
IL-22	Interleukin 22
IPEC	Intestinal pathogenic *Escherichia coli*
LDH	Lactate dehydrogenase
LDL	Low-density lipoproteins
MDA	Malondialdehyde
NAC	*N*-acetyl-*L*-cysteine
PTH	Parathyroid hormone
RBCs	Red blood cells
ROS	Reactive oxygen species
STEC	Shiga toxin-producing *Escherichia coli*
STEC-HUS	Shiga toxin-producing *Escherichia coli*-associated hemolytic uremic syndrome
Stx	Shiga toxins
TAC	Total antioxidant capacity
TMA	Thrombotic microangiopathies
TNF-α	Tumor necrosis factor alpha
TOS	Total oxidative stress
TTP	Thrombotic Thrombocytopenic Purpura

## References

[1] Binks S., Regan K., Richenberg J., Chevassut T. (2012). Microbes without Frontiers: Severe Haemolytic-Uraemic Syndrome Due to E Coli O_104_:H_4_. BMJ Case Rep..

[2] Delmas Y., Vendrely B., Clouzeau B., Bachir H., Bui H.-N., Lacraz A., Helou S., Bordes C., Reffet A., Llanas B. (2014). Outbreak of *Escherichia coli* O_104_:H_4_ Haemolytic Uraemic Syndrome in France: Outcome with Eculizumab. Nephrol. Dial. Transplant..

[3] Joseph A., Rafat C., Zafrani L., Mariani-Kurkdjian P., Veyradier A., Hertig A., Rondeau E., Mariotte E., Azoulay E. (2018). Early Differentiation of Shiga Toxin–Associated Hemolytic Uremic Syndrome in Critically Ill Adults With Thrombotic Microangiopathy Syndromes. Crit. Care Med..

[4] Liu Y., Thaker H., Wang C., Xu Z., Dong M. (2022). Diagnosis and Treatment for Shiga Toxin-Producing *Escherichia coli* Associated Hemolytic Uremic Syndrome. Toxins.

[5] Hunt J.M. (2010). Shiga Toxin–Producing *Escherichia coli* (STEC). Clin. Lab. Med..

[6] Travert B., Rafat C., Mariani P., Cointe A., Dossier A., Coppo P., Joseph A. (2021). Shiga Toxin-Associated Hemolytic Uremic Syndrome: Specificities of Adult Patients and Implications for Critical Care Management. Toxins.

[7] Bruyand M., Mariani-Kurkdjian P., Gouali M., de Valk H., King L.A., Le Hello S., Bonacorsi S., Loirat C. (2018). Hemolytic Uremic Syndrome Due to Shiga Toxin-Producing *Escherichia coli* Infection. Méd. Mal. Infect..

[8] Nasir A., Patel N., Prabakaran S., Sagheer S.H., Troy S.P., Baldinger E., Frontera A. (2019). Hemolytic Uremic Syndrome with Severe Neurologic Complications in an Adult. Fed. Pract..

[9] Loos S., Oh J., van de Loo L., Kemper M.J., Blohm M., Schild R. (2021). Hemoconcentration and Predictors in Shiga Toxin-Producing E. Coli-Hemolytic Uremic Syndrome (STEC-HUS). Pediatr. Nephrol..

[10] Aroor S., Teja Gajjala S., Kini P., Mundkur S., Bhat Y.R., Kumar S. (2024). Hemolytic Uremic Syndrome in Children: Clinical Characteristics and Predictors of Outcome. Clin. Epidemiol. Glob. Health.

[11] Mengistu D.Y., Mengesha Y. (2023). New Approaches for Severity Intervention and Rapid Diagnosis of Enterohemorrhagic Escherichia Coli: A Review. All Life.

[12] Menne J., Nitschke M., Stingele R., Abu-Tair M., Beneke J., Bramstedt J., Bremer J.P., Brunkhorst R., Busch V., Dengler R. (2012). Validation of Treatment Strategies for Enterohaemorrhagic *Escherichia coli* O_104_:H_4_ Induced Haemolytic Uraemic Syndrome: Case-Control Study. BMJ.

[13] Gomez S.A., Abrey-Recalde M.J., Panek C.A., Ferrarotti N.F., Repetto M.G., Mejías M.P., Fernández G.C., Vanzulli S., Isturiz M.A., Palermo M.S. (2013). The Oxidative Stress Induced in Vivo by Shiga Toxin-2 Contributes to the Pathogenicity of Haemolytic Uraemic Syndrome. Clin. Exp. Immunol..

[14] Hsu S.-P., Chiang C.-K., Yang S.-Y., Chien C.-T. (2010). N-Acetylcysteine for the Management of Anemia and Oxidative Stress in Hemodialysis Patients. Nephron Clin. Pract..

[15] Eligini S., Porro B., Aldini G., Colli S., Banfi C. (2022). N-Acetylcysteine Inhibits Platelet Function through the Regeneration of the Non-Oxidative Form of Albumin. Antioxidants.

[16] Chen J., Reheman A., Gushiken F.C., Nolasco L., Fu X., Moake J.L., Ni H., López J.A. (2011). N-Acetylcysteine Reduces the Size and Activity of von Willebrand Factor in Human Plasma and Mice. J. Clin. Investig..

[17] Ye M., Lin W., Zheng J., Lin S. (2021). N-Acetylcysteine for Chronic Kidney Disease: A Systematic Review and Meta-Analysis. Am. J. Transl. Res..

[18] Ramos S., Silva V., Dapkevicius M.d.L.E., Caniça M., Tejedor-Junco M.T., Igrejas G., Poeta P. (2020). *Escherichia coli* as Commensal and Pathogenic Bacteria among Food-Producing Animals: Health Implications of Extended Spectrum β-Lactamase (ESBL) Production. Animals.

[19] Karch H. (2001). The Role of Virulence Factors in Enterohemorrhagic *Escherichia coli* (EHEC)—Associated Hemolytic-Uremic Syndrome. Semin. Thromb. Hemost..

[20] Licznerska K., Nejman-Faleńczyk B., Bloch S., Dydecka A., Topka G., Gąsior T., Węgrzyn A., Węgrzyn G. (2016). Oxidative Stress in Shiga Toxin Production by Enterohemorrhagic Escherichia Coli. Oxidative Med. Cell. Longev..

[21] Wijnsma K.L., Veissi S.T., de Wijs S., van der Velden T., Volokhina E.B., Wagener F.A.D.T.G., van de Kar N.C.A.J., van den Heuvel L.P. (2020). Heme as Possible Contributing Factor in the Evolvement of Shiga-Toxin *Escherichia coli* Induced Hemolytic-Uremic Syndrome. Front. Immunol..

[22] Ullah I., Lang M. (2023). Key Players in the Regulation of Iron Homeostasis at the Host-Pathogen Interface. Front. Immunol..

[23] Suits M.D.L., Pal G.P., Nakatsu K., Matte A., Cygler M., Jia Z. (2005). Identification of an *Escherichia coli* O157:H7 Heme Oxygenase with Tandem Functional Repeats. Proc. Natl. Acad. Sci. USA.

[24] Garénaux A., Caza M., Dozois C.M. (2011). The Ins and Outs of Siderophore Mediated Iron Uptake by Extra-Intestinal Pathogenic Escherichia Coli. Vet. Microbiol..

[25] Contreras H., Chim N., Credali A., Goulding C.W. (2014). Heme Uptake in Bacterial Pathogens. Curr. Opin. Chem. Biol..

[26] Fox D.R., Asadollahi K., Samuels I., Spicer B.A., Kropp A., Lupton C.J., Lim K., Wang C., Venugopal H., Dramicanin M. (2025). Inhibiting Heme Piracy by Pathogenic *Escherichia coli* Using de Novo-Designed Proteins. Nat. Commun..

[27] Nagy G., Dobrindt U., Kupfer M., Emödy L., Karch H., Hacker J. (2001). Expression of Hemin Receptor Molecule ChuA Is Influenced by RfaH in Uropathogenic *Escherichia coli* Strain 536. Infect. Immun..

[28] Wagener F.A.D.T.G., van de Kar N.C.A.J., van den Heuvel L.P. (2022). Protective Mechanisms Harnessing against Injurious Heme and Preventing Kidney Damage in STEC-HUS: Toward New Therapies?. Kidney Int..

[29] Zoja C., Buelli S., Morigi M. (2010). Shiga Toxin-Associated Hemolytic Uremic Syndrome: Pathophysiology of Endothelial Dysfunction. Pediatr. Nephrol..

[30] Chan Y.S., Ng T.B. (2016). Shiga Toxins: From Structure and Mechanism to Applications. Appl. Microbiol. Biotechnol..

[31] Menge C. (2020). Molecular Biology of *Escherichia coli* Shiga Toxins’ Effects on Mammalian Cells. Toxins.

[32] Mittal M., Siddiqui M.R., Tran K., Reddy S.P., Malik A.B. (2014). Reactive Oxygen Species in Inflammation and Tissue Injury. Antioxid. Redox Signal..

[33] Larsen R., Gouveia Z., Soares M.P., Gozzelino R. (2012). Heme Cytotoxicity and the Pathogenesis of Immune-Mediated Inflammatory Diseases. Front. Pharmacol..

[34] Belcher J.D., Beckman J.D., Balla G., Balla J., Vercellotti G. (2010). Heme Degradation and Vascular Injury. Antioxid. Redox Signal..

[35] Chiabrando D., Vinchi F., Fiorito V., Mercurio S., Tolosano E. (2014). Heme in Pathophysiology: A Matter of Scavenging, Metabolism and Trafficking across Cell Membranes. Front. Pharmacol..

[36] Stefanova D., Raychev A., Deville J., Humphries R., Campeau S., Ruchala P., Nemeth E., Ganz T., Bulut Y. (2018). Hepcidin Protects against Lethal *Escherichia coli* Sepsis in Mice Inoculated with Isolates from Septic Patients. Infect. Immun..

[37] Obrig T.G., Karpman D. (2011). Shiga Toxin Pathogenesis: Kidney Complications and Renal Failure. Ricin and Shiga Toxins: Pathogenesis, Immunity, Vaccines and Therapeutics.

[38] Imlay J.A. (2013). The Molecular Mechanisms and Physiological Consequences of Oxidative Stress: Lessons from a Model Bacterium. Nat. Rev. Microbiol..

[39] Long N., Deng J., Qiu M., Zhang Y., Wang Y., Guo W., Dai M., Lin L. (2022). Inflammatory and Pathological Changes in *Escherichia coli* Infected Mice. Heliyon.

[40] Aiassa V., Baronetti J.L., Paez P.L., Barnes A.I., Albrecht C., Pellarin G., Eraso A.J., Albesa I. (2011). Increased Advanced Oxidation of Protein Products and Enhanced Total Antioxidant Capacity in Plasma by Action of Toxins of *Escherichia coli* STEC. Toxicol. In Vitro.

[41] Exeni R.A., Fernandez-Brando R.J., Santiago A.P., Fiorentino G.A., Exeni A.M., Ramos M.V., Palermo M.S. (2018). Pathogenic Role of Inflammatory Response during Shiga Toxin-Associated Hemolytic Uremic Syndrome (HUS). Pediatr. Nephrol..

[42] Vallelian F., Buehler P.W., Schaer D.J. (2022). Hemolysis, Free Hemoglobin Toxicity, and Scavenger Protein Therapeutics. Blood.

[43] Arvidsson I., Ståhl A., Manea Hedström M., Kristoffersson A.-C., Rylander C., Westman J.S., Storry J.R., Olsson M.L., Karpman D. (2015). Shiga Toxin–Induced Complement-Mediated Hemolysis and Release of Complement-Coated Red Blood Cell–Derived Microvesicles in Hemolytic Uremic Syndrome. J. Immunol..

[44] Sakamoto K., Kim Y.-G., Hara H., Kamada N., Caballero-Flores G., Tolosano E., Soares M.P., Puente J.L., Inohara N., Núñez G. (2017). IL-22 Controls Iron-Dependent Nutritional Immunity against Systemic Bacterial Infections. Sci. Immunol..

[45] Rushworth G.F., Megson I.L. (2014). Existing and Potential Therapeutic Uses for N-Acetylcysteine: The Need for Conversion to Intracellular Glutathione for Antioxidant Benefits. Pharmacol. Ther..

[46] Aruoma O.I., Halliwell B., Hoey B.M., Butler J. (1989). The Antioxidant Action of N-Acetylcysteine: Its Reaction with Hydrogen Peroxide, Hydroxyl Radical, Superoxide, and Hypochlorous Acid. Free Radic. Biol. Med..

[47] PubChem: N-Acetyl-L-Cysteine Compound Summary. https://pubchem.ncbi.nlm.nih.gov/compound/N-Acetyl-L-Cysteine.

[48] Olsson B., Johansson M., Gabrielsson J., Bolme P. (1988). Pharmacokinetics and Bioavailability of Reduced and Oxidized N-Acetylcysteine. Eur. J. Clin. Pharmacol..

[49] Shetty R., Udupa N., Mutalik S., Kulkarni V., Rao V. (2019). Mechanisms and Therapeutics of N-Acetylcysteine: A Recent Update. Res. J. Pharm. Technol..

[50] Aldini G., Altomare A., Baron G., Vistoli G., Carini M., Borsani L., Sergio F. (2018). N-Acetylcysteine as an Antioxidant and Disulphide Breaking Agent: The Reasons Why. Free Radic. Res..

[51] Atkuri K.R., Mantovani J.J., Herzenberg L.A., Herzenberg L.A. (2007). N-Acetylcysteine-a Safe Antidote for Cysteine/Glutathione Deficiency. Curr. Opin. Pharmacol..

[52] Winterbourn C.C. (1995). Toxicity of Iron and Hydrogen Peroxide: The Fenton Reaction. Toxicol. Lett..

[53] Gutteridge J.M.C. (1992). Iron and Oxygen Radicals in Brain. Ann. Neurol..

[54] Ferraris V., Acquier A., Ferraris J.R., Vallejo G., Paz C., Mendez C.F. (2011). Oxidative Stress Status during the Acute Phase of Haemolytic Uraemic Syndrome. Nephrol. Dial. Transplant..

[55] Tenório M.C.D.S., Graciliano N.G., Moura F.A., de Oliveira A.C.M., Goulart M.O.F. (2021). N-Acetylcysteine (NAC): Impacts on Human Health. Antioxidants.

[56] Hernández-Cruz E.Y., Aparicio-Trejo O.E., Hammami F.A., Bar-Shalom D., Tepel M., Pedraza-Chaverri J., Scholze A. (2024). N-Acetylcysteine in Kidney Disease: Molecular Mechanisms, Pharmacokinetics, and Clinical Effectiveness. Kidney Int. Rep..

[57] Ahmed H.M., Maksoud F.A.W.A., Sayed A.R., Hussein R.R.S., Abdelwahed A., Ramadan Y.M. (2025). N-Acetylcysteine for Treatment of Anemia in Children with Kidney Failure: A Prospective Study. Ren. Replace. Ther..

[58] Shajari R., Zavar Reza J., Najafi F., Hemayati R. (2024). The Effect of N-Acetylcysteine on Inflammatory and Oxidative Markers in Patients Receiving Hemodialysis; a Single-Arm Clinical Trial Study. J. Nephropharmacol..

[59] Bashardoust B., Alaei R., Mohammadi Kebar S., Hasani S., Habibzadeh A. (2017). The Effect of Oral N-Acetylcysteine on Serum High Sensitive CRP and Plasma Hemoglobin Levels in End-Stage Renal Disease Patients under Routine Hemodialysis; a Randomized Placebocontrolled Clinical Trial. J. Nephropathol..

[60] Schwalfenberg G.K. (2021). N-Acetylcysteine: A Review of Clinical Usefulness (an Old Drug with New Tricks). J. Nutr. Metab..

[61] Lee D., Bae C., Ryu S., Lee K. (2024). N-Acetyl Cysteine-loaded Liposomes to Reduce Iron Overload-induced Toxicity in Human Kidney Cells. J. Chem. Technol. Biotechnol..

[62] Sharma P., Kumar R., Bari A., Singh S.K. (2025). N-Acetyl Cysteine and Vitamin C Modulate the Antibiotic Efficacy Against *Escherichia coli* Cells. Microb. Drug Resist..

[63] Manoharan A., Ognenovska S., Paino D., Whiteley G., Glasbey T., Kriel F.H., Farrell J., Moore K.H., Manos J., Das T. (2021). N-Acetylcysteine Protects Bladder Epithelial Cells from Bacterial Invasion and Displays Antibiofilm Activity against Urinary Tract Bacterial Pathogens. Antibiotics.

[64] Chlumsky O., Smith H.J., Parker A.E., Brileya K., Wilking J.N., Purkrtova S., Michova H., Ulbrich P., Viktorova J., Demnerova K. (2021). Evaluation of the Antimicrobial Efficacy of N-Acetyl-l-Cysteine, Rhamnolipids, and Usnic Acid—Novel Approaches to Fight Food-Borne Pathogens. Int. J. Mol. Sci..

[65] Clemente E., Della Corte M., Ferrara M., Cerchia E., Catti M., Garazzino S., Gerocarni Nappo S., Bonora S. (2024). N-Acetylcysteine’s Potential Role in Prophylaxis and Treatment of Pediatric Urinary Tract Infections: From Evidence to Patient-Side Research. Surgeries.

[66] Wang H.-Z., Peng Z.-Y., Wen X.-Y., Rimmelé T., Bishop J.V., Kellum J.A. (2011). N-Acetylcysteine Is Effective for Prevention but Not for Treatment of Folic Acid-Induced Acute Kidney Injury in Mice. Crit. Care Med..

[67] Demirci U., Bilgin Z.T., Baysal M. (2026). N-Acetylcysteine Therapy in Thrombotic Thrombocytopenic Purpura: A Systematic Review and Critical Appraisal. J. Clin. Med..

[68] Jalbert A., Yao H., Fagnan M., Crevier B. (2023). Eculizumab in the Treatment of Acetylcysteine-Induced Atypical Hemolytic Uremic Syndrome. Cureus.

